# Transplantation of Neural Stem Cells Cotreated with Thyroid Hormone and GDNF Gene Induces Neuroprotection in Rats of Chronic Experimental Allergic Encephalomyelitis

**DOI:** 10.1155/2016/3081939

**Published:** 2016-01-10

**Authors:** Xiaoqing Gao, Guangqiang Hu, Li Deng, Guangbi Fan, Chaoxian Yang, Jie Du

**Affiliations:** Department of Anatomy and Neurobiology, Sichuan Medical University, No. 319, Zhongshan Road, Luzhou, Sichuan 646000, China

## Abstract

The present study investigates whether transplantation of NSCs treated with T3 alone (T3/NSCs), or in conjunction with GDNF gene (GDNF-T3/NSCs), provides a better therapeutic effect than NSCs for chronic EAE. EAE rats were, respectively, injected with NSCs, T3/NSCs, GDNF-T3/NSCs, and saline at 10 days and sacrificed at 60 days after EAE immunization. The three cell grafted groups showed a significant reduction in clinical scores, inflammatory infiltration, and demyelination compared with the saline-injected group, and among the cell grafted groups, the reduction in GDNF-T3/NSCs group was the most notable, followed by T3/NSCs group. Grafted T3/NSCs and GDNF-T3/NSCs acquired more MAP2, GalC, and less GFAP in brain compared with grafted NSCs, and grafted GDNF-T3/NSCs acquired most MAP2 and least GalC among the cell grafted groups. Furthermore, T3/NSCs and GDNF-T3/NSCs grafting increased the expression of mRNA for PDGF*α*R, GalC, and MBP in lesion areas of brain compared with NSCs grafting, and the expression of mRNA for GalC and MBP in GDNF-T3/NSCs group was higher than that in T3/NSCs group. In conclusion, T3/NSCs grafting, especially GDNF-T3/NSCs grafting, provides a better neuroprotective effect for EAE than NSCs transplantation.

## 1. Introduction

Chronic experimental allergic encephalomyelitis (EAE), an animal model of multiple sclerosis (MS), is an autoimmune disease in the CNS characterized by extensive plaques or lesions throughout grey and white matter, with loss of myelin and myelinating cells as well as damage to axons and neurons [1]. The general failure of endogenous remyelination and inflammation-related axonal damage lead to accumulated neurological disability [2]. The current immunomodulating drugs for MS, such as interferon *β*, glatiramer acetate, and natalizumab, prevent inflammatory damage to the CNS, which do not effectively prevent the progressive clinical neurological decline most likely because they lack a direct action on CNS axons and myelin [3]. Neural stem cells (NSCs), which can differentiate into various neural cell types [2, 4] and have myelinogenic potential [5] in the injured areas, have been introduced as a promising cell type of replacement therapy. Driving neurons and oligodendrocytes commitment and development of transplanted NSCs should largely promote the success of transplantation therapy for EAE.

Thyroid hormone (T3) is required for production and maturation of oligodendrocytes and proper myelination [6, 7]. T3 supplementation enhances remyelination, axon protection, and nerve conduction preservation in various animal demyelinating models [8–10]. Moreover, NSCs from EAE animals show a strong limitation in oligodendrocyte generation, which is completely recovered by T3 treatment [7]. Glial cell line-derived neurotrophic factor (GDNF) has been proved to have a potent neuroprotective effect on various neuronal damage [11–13] and also has an ability to promote axon regeneration and myelination after spinal cord injury [14]. GDNF gene modified-fibroblasts transplantation promoted significant axons regeneration and remyelination of regenerating axons following spinal cord transection injury [15]. Additionally, our previous studies also showed that GDNF gene modified-NSCs provided more efficient neuroprotection for stroke rats than native NSCs [16]. In the present study, we investigated whether transplantation of NSCs cotreated with T3 and GDNF provides better neuroprotective effect than transplantation of native NSCs for EAE.

## 2. Materials and Methods

### 2.1. Growth, Induction, and Infection of Rat Neurospheres

The cerebral hemispheres dissected from newborn Wistar rats (Animal House Center, Sichuan Medical University, Sichuan, China) were minced and dissociated into single cell suspensions. The cells were suspended in 25 mL flask at a density of 1 × 10^5^ cells/mL in stem medium (serum-free DMEM/F12 medium supplemented with 20 ng/mL of basic fibroblast growth factor (bFGF), 20 ng/mL of epidermal growth factor (EGF), and 1% N2 supplement (all from Gibco, USA)). After 5~7 days of culture, neurospheres were formed and assayed for nestin expression by immunohistochemical staining. Then neurospheres were triturated into single cells suspension. The detached NSCs were divided into two groups. One group was planted in stem medium additionally added 20 ng/mL of T3 for 5 days (T3/NSCs); the other was infected with GDNF recombinant adenovirus (pAdEasy-1-pAdTrack CMV-GDNF) for 2 days as described in our laboratory [16]. Adeasy-1 plasmid contains the gene for green fluorescent protein and the titers of viral were 1 × 10^9^ PFU/mL. Briefly, 200 *μ*L single cell suspensions mixing 5 *μ*L GDNF recombinant adenovirus solution were planted in each well of a 24-well plate in 5% CO_2_ at 37°C for 2 hours; then 300 *μ*L medium was added to each well to further incubate for 2 days. The postinfected cells were then planted in stem medium containing 20 ng/mL of T3 for 5 days (GDNF-T3/NSCs). For labeling in vivo the grafted cells, NSCs, T3/NSCs, and GDNF-T3/NSCs were pretreated for 3 days prior to grafting with 10 *μ*M of 5-bromo-2′-deoxyuridine (BrdU, Sigma, USA).

### 2.2. EAE Induction and Neurospheres Transplantation

All animal experiments were approved by and performed in accordance with the Chinese Academy of Sciences, China. EAE is induced in female Wistar rats by guinea pig spinal cord homogenate emulsified with complete Freund's adjuvant (CFA, Sigma). 6–8-week-old female Wistar rats (inbred strain, Animal House Center, Sichuan Medical University, Sichuan, China) were injected in the four footpads of 100 mg of guinea pig spinal cord in 0.4 mL of PBS emulsified with equal volume of CFA supplemented with 6 mg/mL of mycobacterium tuberculosis H37Ra (Shijiazhuang Weitian Scientific Instruments Equipment Co., Ltd., China). At day of induction and at day 2, 300 ng of bordetella pertussis toxin (Weitian) in 0.1 mL PBS was injected subcutaneously. The animals were scored daily for neurological symptoms as follows: (0) no clinical disease; (1) limpness in tail; (2) hind-leg ataxia; (3) hind-leg paralysis; (4) paraplegia; and (5) moribund or dead. Ten days after EAE induction, rats were anesthetized with intraperitoneal injection of pentobarbital sodium (30 mg/kg) and were fixed on a stereotactic device (Angle Two Stereotaxic Instrument w/Rat Atlas Product: #464601, USA). Quantities of 5 × 10^5^ cells (NSCs, T3/NSCs, and GDNF-T3/NSCs) in a volume of 10 *μ*L were injected once into each lateral ventricle (AP = −0.8 to 1.0 mm, *R* or *L* = −1.8 to 2.0 mm, and *V* = −4.0 to 5.0 mm). The control group underwent the same injection with 10 *μ*L saline into each lateral ventricle.

### 2.3. Histological and Immunohistochemical Assessment

EAE rats were sacrificed with a lethal dose of pentobarbital sodium at day 60 after EAE induction. 20 rats (5 per group) were perfused via the ascending aorta with PBS followed by 4% paraformaldehyde. The brains were removed and postfixed for 24 hours by immersion in the same fixative. Histopathology and immunohistochemistry were assessed in 6 *μ*m thick paraffin sections at 0.2~1.6 mm below the bregma levels. Hematoxylin-eosin (HE) staining was used to evaluate inflammatory infiltration. 10 nuclei or more gathered in cerebrum white matter or surrounded by a blood vessel were considered as an infiltration lesion. To estimate the extent of inflammation, the number of infiltration lesions and the number of cells per infiltration lesion were counted. Luxol fast blue (LFB, Sigma) staining was performed to detect the degree of demyelination and the measurement parameter was the integrated optical density (IOD).

BrdU and neural special markers double immunohistochemistry staining were used to identify grafted cells and the differentiated cells from the grafted cells in vivo. The special markers were neuronal specific markers microtubule-associated protein 2 (rabbit anti-MAP2, 1 : 100, Abcam, UK), astrocytes specific markers glial fibrillary acidic protein (rabbit anti-GFAP, 1 : 100, Abcam), and oligodendrocyte specific marker galactocerebroside (rabbit anti-GalC, 1 : 50, Chemicon, USA). Briefly, sections were incubated in 0.3% H_2_O_2_ in methanol for 10 minutes, followed by incubation with 0.1% Triton X-100 in 0.1% sodium citrate for 10 minutes. The sections were treated with 2 N HCl at 37°C for 1 hour and then incubated with mouse anti-BrdU (1 : 200, Abcam) at 4°C overnight. After a goat anti-mouse IgG secondary antibody (1 : 100; Wuhan Boster Biological Technology, China) was added for 30 minutes, alkaline phosphatase- (AP-) streptavidin (Boster) was incubated for 30 minutes, and 5-bromo-4-chloro-3-indolyl phosphate/nitroblue tetrazolium chloride (BCIP/NBT, Boster) was then used as a chromogen for 10 min. After PBS washes, the sections were reincubated with primary antibody for rabbit ant-MAP2, GFAP, or GalC, followed by incubation with goat anti-rabbit IgG (1 : 100; Boster) and horseradish peroxidase- (HRP-) streptavidin (Boster). Diaminobenzidine (DAB, Boster) or 3-amino-9-ethylcarbazole (AEC, Boster) was then used as a chromogen for light microscopy. Negative control sections from each animal were carried out, except that the primary antibody was omitted.

IOD was counted in corpus callosum in 3 sections per animal using ImageJ 1.44p software. Inflammatory infiltration and positive cells were assessed in 6 regions of interest situated within bilaterally white matter tracts in 3 sections per animal. Measurement was made in a predefined field (0.6 mm × 0.6 mm) using image-pro plus 6.0 software. The percentage of MAP2^+^, GFAP^+^, and GalC^+^ cells was quantified by normalizing total MAP2^+^, GFAP^+^, or GalC^+^ cells to the total number of BrdU^+^ cells.

### 2.4. Reverse Transcription-PCR Analysis

For quantitative assessments of relative mRNA levels, total RNA from brains of 20 rats (5 per group) was extracted using the TRIZOL reagent (Invitrogen, USA), following the manufacturer's instructions. 2 *μ*g of RNA was reverse transcribed into cDNA using a Takara RNA PCR kit (Takara Biotechnology, Dalian, China). The reaction conditions were 30°C for 10 minutes, 42°C for 30 minutes, 99°C for 5 minutes, and 5°C for 5 minutes. cDNA was subsequently amplified by PCR with specific primers. Sequences of the primers, length of the products, and the annealing temperatures were described in Table 1. Amplification included one stage of 2 minutes at 94°C followed by 30 cycles of three-step loop: 30 seconds at 94°C, 30 seconds at appropriate annealing temperatures, and 1 minute at 72°C. RT-PCR products were analysed by 2% agarose gel electrophoresis and stained by ethidium bromide. The ratio of platelet-derived growth factor *α* receptor (PDGF*α*R), GalC, and myelin basic protein (MBP) to glyceraldehyde-3-phosphate dehydrogenase (GAPDH) was calculated as the relative level of mRNA expression.

### 2.5. Statistical Analysis

All data were presented as mean ± SD. Statistical analysis was performed using SPSS (version 17.0). Group differences were compared using one-way ANOVA, followed by post hoc tests. *P* values < 0.05 were considered statistically significant.

## 3. Results

### 3.1. The Culture and Infection of NSCs

**Figure 1:**
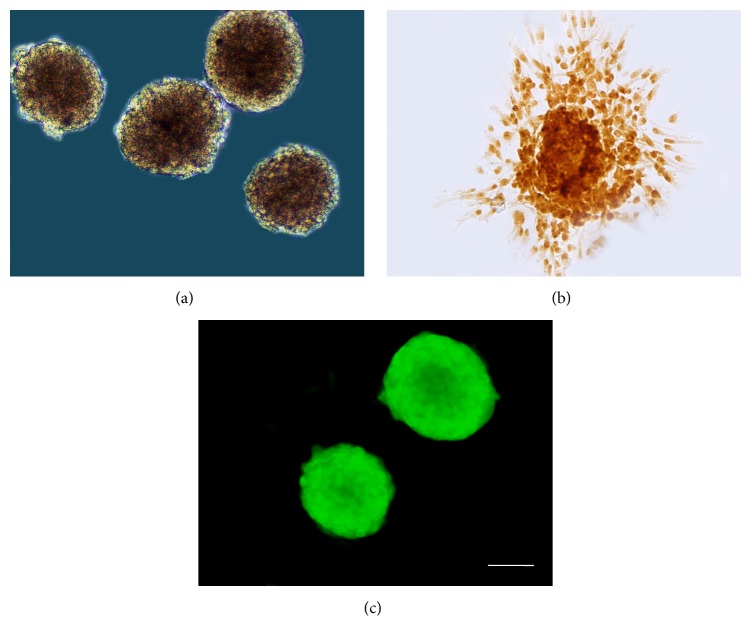
Characterization of the neurosphere prior to transplantation. (a) Neurospheres derived from cerebral parenchyma of newborn rats for 7 days. (b) Immunocytochemical staining of nestin in neurospheres. (c) Neurospheres infected with GDNF recombinant adenovirus on launching green fluorescence. Bar (a–c) = 75 *μ*m.

NSCs were isolated from newborn Wistar rats cerebral hemispheres and proliferated into round floating spheres after 5~7 days of culture (Figure 1(a)) as nestin-positive cells (Figure 1(b)). NSCs infected by GDNF recombinant adenovirus for 2 days showed green fluorescence under fluorescence microscope (Figure 1(c)).

### 3.2. Attenuation of Clinical Disease following Neurospheres Transplantation

**Figure 2:**
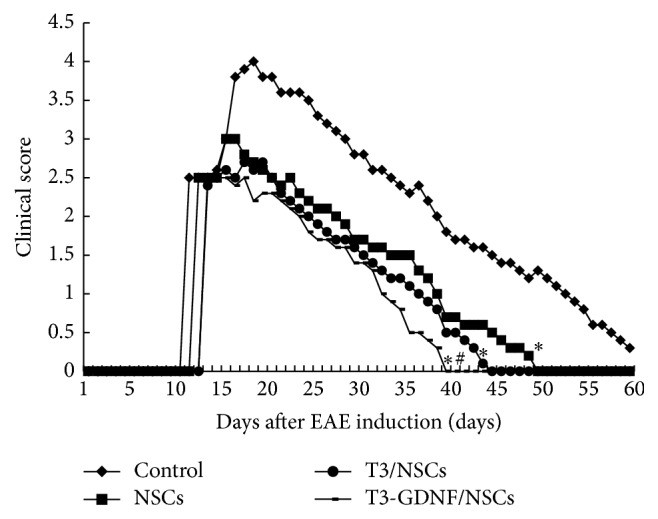
Clinical severity improvement following neurosphere transplantation (mean ± SD, *n* = 5). ^*∗*^Comparison with control group, *P* < 0.05; ^#^comparison with NSCs group, *P* < 0.05.

To evaluate the effects of NSCs on disease progression in EAE, neurospheres (NSCs, T3/NSCs and GDNF-T3/NSCs) were transplanted into lateral ventricles at day 10 after EAE induction. Disabilities appeared typically in each group at days 11~14 after EAE induction with an average clinical score of 2.3~2.5, reaching to the maximum score after 6~7 days. The animals in three neurosphere-grafted groups showed clinical symptoms rapidly reduced compared to saline-injected animals (*P* < 0.05) and recovered normal gait after 40~50 days of induction, whereas the saline-injected EAE rats still had slight symptoms at the end of follow-up period (Figure 2). Moreover, the improvement of clinical symptoms in GDNF-T3/NSCs group was the most notable among the grafted groups (Figure 2).

### 3.3. Reduction of Brain Inflammation and Demyelinating Process by Neurospheres Transplantation

**Figure 3:**
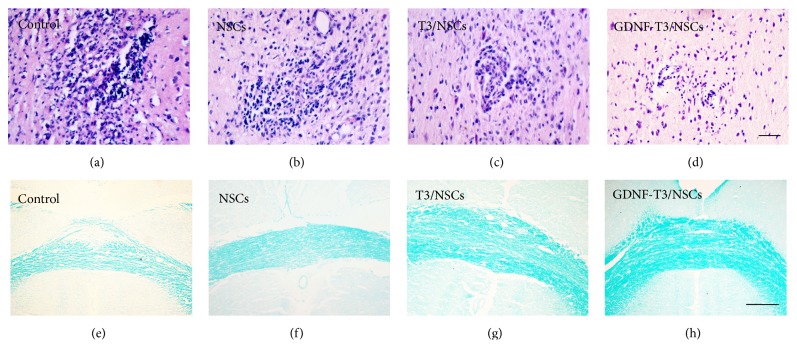
HE staining of inflammatory lesions of the cerebral parenchyma and LFB staining of demyelination of the corpus callosum in control, NSCs, T3/NSCs, and GDNF-T3/NSCs groups, respectively. (a) Control group showed much more inflammatory cell infiltrations. ((b), (c), and (d)) Neurosphere-treated groups displayed reduced number and size of inflammatory cell infiltrations, especially in GDNF-T3/NSCs groups (d). (e) Control group showed marked myelin sparse and loss. ((f), (g), and (h)) Neurosphere-treated groups displayed a preservation of the myelin structure, especially in GDNF-T3/NSCs groups (h). (a–d) = 50 *μ*m, (e–h) = 100 *μ*m.

To assess if NSCs transplantation attenuates brain inflammation and demyelination, HE staining was used to evaluate the number and size of infiltration lesions. Luxol fast blue (LFB) staining was used to assess the demyelination. Saline-injected rats showed much more typical inflammatory cell infiltration (Figure 3(a)) and marked myelin sparse and loss (Figure 3(e)). In contrast, neurosphere-treated animals displayed reduced number and size of inflammatory cell infiltrations (Figures 3(b)–3(d), Table 2) concomitant with a preservation of the myelin structure and an increase of myelin density (Figures 3(f)–3(h), Table 2), indicating that saline-injected EAE rats experienced progressive inflammation and demyelination, which was blocked by neurospheres transplantation. No significant differences in the mean number of infiltration lesions were observed between the NSCs and the T3/NSCs groups; however, the mean number of cells per infiltrate in the T3/NSCs groups was less than that in NSCs group (*P* < 0.05), and the myelin structure was also more perfect and myelin density was higher than that of NSCs group (*P* < 0.05). Additionally, the mean number of infiltration lesions and mean number of cells per infiltrate in GDNF-T3/NSCs group were apparently less than those in T3/NSCs group (*P* < 0.05), and the myelin structure in GDNF-T3/NSCs group was also most complete and myelin density was highest among the transplanted groups (*P* < 0.05). The above results showed GDNF-T3/NSCs had the strongest improvement in inflammation and demyelination for EAE, and T3/NSCs were stronger than NSCs.

### 3.4. Integration of Transplanted Neurospheres in the Host EAE Brain

**Figure 4:**
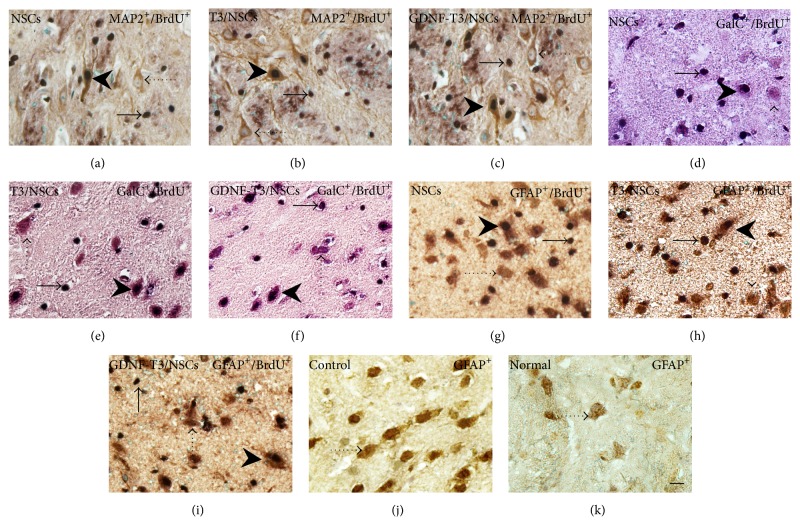
Immunohistochemical double staining of BrdU, MAP2, GFAP, and GalC positive cells in the cerebral parenchyma of rats. (a–c) MAP2^−^/BrdU^+^ cells (solid arrow), MAP2^+^/BrdU^−^ cells (dotted arrow), and MAP2^+^/BrdU^+^ cells (arrowhead) in NSCs, T3/NSCs, and GDNF-T3/NSCs group, respectively. (d–f) GalC^−^/BrdU^+^ positive cells (solid arrow), GalC^+^/BrdU^−^ cells (dotted arrow), and GalC^+^/BrdU^+^ cells (arrowhead) in NSCs, T3/NSCs, and GDNF-T3/NSCs group, respectively. (g–i) GFAP^−^/BrdU^+^ cells (solid arrow), GFAP^+^/BrdU^−^ cells (dotted arrow), and GFAP^+^/BrdU^+^ cells (arrowhead) in NSCs, T3/NSCs, and GDNF-T3/NSCs group, respectively. (j) GFAP^+^ cells (dotted arrow) in control group. (k) GFAP^+^ cells (dotted arrow) in normal group. Bar (a–k) = 25 *μ*m.

The transplanted cells at 60 days after EAE induction disseminated predominantly in the inflamed areas. No significant differences in the density of BrdU-positive cells per mm^2^ in brain between NSCs and T3/NSCs groups were observed (*P* > 0.05). The density of BrdU-positive cells per mm^2^ in the GDNF-T3/NSCs group was significantly higher (*P* < 0.05) than that in the NSCs and T3/NSCs groups, which suggested treatment of GDNF gene raised grafted NSCs survival in EAE brain. Double immunohistochemistry showed that transplanted T3/NSCs and GDNF-T3/NSCs acquired more MAP2 and GalC and fewer GFAP than grafted NSCs (*P* < 0.05), and the GDNF-T3/NSCs group had the highest MAP2 and lowest GFAP among the three groups of cells transplantation (*P* < 0.05) (Figures 4(a)–4(i), Table 3).

### 3.5. Inhibition of Endogenous Astrocytes Proliferation by Neurospheres Transplantation

Astrogliosis induced all perturbations of the CNS environment virtually, and it has become a pathological hallmark of CNS structural lesions. In order to determine the effect of neurospheres transplantation on proliferation of endogenous astrocytes, GFAP^+^/BrdU^−^ cells were counted in the grafted rats, and GFAP^+^ cells in normal and control rats were also counted as controls. All the EAE rats showed significantly increased expression of endogenous astrocytes in lesion areas compared with normal rats (*P* < 0.05). The EAE rats receiving neurospheres transplantation, however, showed reduced number of endogenous astrocytes compared with the EAE rats receiving saline injection (*P* < 0.05). Among the three groups of cells transplantation, GDNF-T3/NSCs group had the lowest proliferation (*P* < 0.05), and T3/NSCs group was lower than NSCs group (*P* < 0.05) (Figures 4(g)–4(k), Table 4).

### 3.6. Increase of mRNA of Oligodendrocyte Lineage Cells after Neurospheres Transplantation

**Figure 5:**
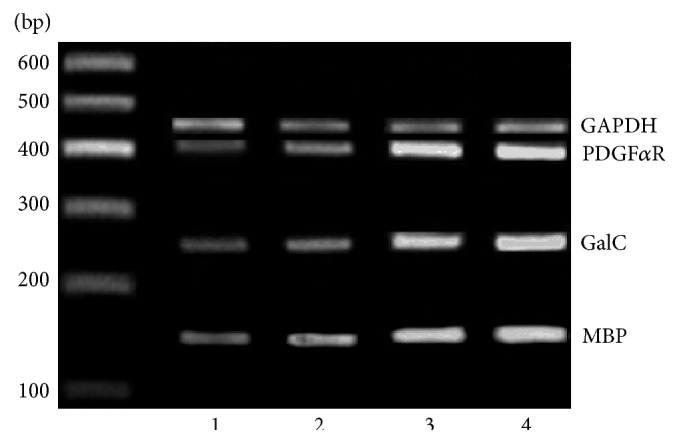
Expression of mRNA for PDGF*α*R, GalC, and MBP in the cerebral lesion areas in each group. 1: control group; 2: NSCs group; 3: T3/NSCs group; 4: GDNF-T3/NSCs group.

Cells grafting significantly increased the expression of mRNA for PDGF*α*R, GalC, and MBP (the markers of oligodendrocyte lineage cells) in cerebral lesion areas compared with saline-injected control EAE rats (*P* < 0.05). The three mRNA expressions in T3/NSCs and GDNF-T3/NSCs groups were higher than that in NSCs group (*P* < 0.05); the expression of mRNA for GalC and MBP in GDNF-T3/NSCs group was higher than that in T3/NSCs group (*P* < 0.05) (Figure 5, Table 5). The results suggested T3/NSCs grafting, especially GDNF-T3/NSCs grafting, promotes oligodendrogenesis and remyelination for EAE rats compared to NSCs grafting.

## 4. Discussion

Our study shows that grafting NSCs, T3/NSCs, and GDNF-T3/NSCs into rats subjected to EAE significantly improved functional outcome compared with control group. T3/NSCs and GDNF-T3/NSCs groups reduced significantly inflammatory infiltrations and demyelination compared with NSCs group, and greater reductions in GDNF-T3/NSCs group were observed compared with the T3/NSCs group. T3/NSCs and GDNF-T3/NSCs generated more MAP2 and GalC positive cells and less GFAP positive cells compared with native NSCs in EAE rats, and more MAP2 and less GFAP positive cells were generated from GDNF-T3/NSCs than T3/NSCs. Moreover, the number of endogenous astrocytes in T3/NSCs and GDNF-T3/NSCs groups was less than that in NSCs group, and between T3/NSCs and GDNF-T3/NSCs groups, GDNF-T3/NSCs group was less than T3/NSCs group. T3/NSCs and GDNF-T3/NSCs grafting significantly increased the expression of mRNA for PDGF*α*R, GalC, and MBP in lesion areas of brain, and the expression of mRNA for GalC and MBP in GDNF-T3/NSCs group was higher than that in T3/NSCs group. Furthermore, the number of transplanted cells in host brain in GDNF-T3/NSCs group was significantly more compared with T3/NSCs and NSCs groups.

EAE is believed to be initiated by T cell-mediated immune responses to myelin antigens [17]. EAE begins when peripherally activated myelin-reactive T cells infiltrate the CNS. Once inside the CNS, myelin-reactive T cells become activated upon exposure to myelin antigens and secrete inflammatory cytokines, thereby triggering an inflammatory cascade that leads to extensive demyelination and neuron/axonal loss [18]. Transplanted NSCs could steadily restrain the expansion of antigen-specific encephalitogenic T cells in lymph nodes of EAE mice, therefrom reducing immune cell mobilization from the periphery [19, 20]. And transplanted NSCs induced apoptosis of blood-borne CNS-infiltrating encephalitogenic T cells in perivascular CNS areas [21]. Previous studies showed that NSCs transplantation ameliorated the clinical signs and reduced tissue injury after EAE, which was associated with reducing the number of perivascular infiltrates and of encephalitogenic T cells in brain of EAE animals [22–24]. Here we showed a significant reduction in the number of inflammatory infiltrates in brain after neurospheres grafting, and the size of infiltration lesions in T3/NSCs and GDNF-T3/NSCs groups was smaller than that in NSCs group; the size in GDNF-T3/NSCs group was the smallest among all the groups. The results suggest that T3/NSCs, especially GDNF-T3/NSCs, more effectively reduce CNS-infiltrating inflammatory cells, thus exerting better effect than NSCs on EAE.

Astrocytes respond to CNS insults with a process of cellular activation known as reactive astrogliosis [25]. Astrocytes activation plays an important role in demyelinating disorders [20, 26–28]. Activated astrocytes orchestrate CNS inflammatory response by producing proinflammatory factors such as interleukin-1*β*, interleukin-6, tumor necrosis factor-*α* [29], and chemokines like CC chemokine ligand-2 [30, 31]. Furthermore, astrogliosis hampers endogenous axonal remyelination, resulting in irreversible axonal loss and functional disability [20, 22]. NSCs transplantation greatly reduced astrogliosis in EAE [22]. Here we verified that proliferation of astrocytes was reduced significantly after neurospheres transplantation. Moreover, among the grafted groups, GDNF-T3/NSCs group had the least proliferation, and T3/NSCs group was less than NSCs group. The results suggested T3/NSCs, especially GDNF-T3/NSCs, had more potent antiastrogliosis capacity than NSCs, thus more profoundly suppressing inflammatory reaction and reducing demyelination than NSCs.

Demyelination and neuronal degeneration in EAE promote disease evolution to the progressive form; thus the therapeutic strategy on EAE still includes mechanisms protecting neurons and repairing demyelination [32]. Newly generated oligodendrocyte lineage cells and neurons from transplanted NSCs markedly decreased the extent of demyelination and promoted axonal regeneration through replacing lost or degenerative cells [1, 22]. In addition, under pathological environments in the CNS, transplanted NSCs differentiate into astrocytes in vivo, which potentially cause reactive gliosis [33, 34]. Thus, it is essential to reduce astrocyte differentiation of NSCs in EAE. Present results showed transplanted T3/NSCs and GDNF-T3/NSCs generated more neurons and oligodendrocytes, and less astrocytes than transplanted NSCs, and transplanted GDNF-T3/NSCs generated most neurons and fewest astrocytes. By contrast, the rats treated with T3/NSCs and GDNF-T3/NSCs had better clinical recovery and less inflammatory activity and demyelination than the rats treated with NSCs, especially the rats treated with GDNF-T3/NSCs.

PDGF*α*R, which is expressed by oligodendrocyte progenitor cells, GalC, which is expressed by immature and mature oligodendrocytes, and MBP, which is expressed by mature myelinating oligodendrocytes, were strongly diminished at the chronic phase of EAE [6, 35, 36]. In this study, NSCs grafting increased the expression of mRNA for PDGF*α*R, GalC, and MBP in lesion areas of brain, the expression in T3/NSCs and GDNF-T3/NSCs groups was higher than NSCs group, and GDNF-T3/NSCs group had the highest mRNA for GalC and MBP. The results suggested that a great number of oligodendrocyte lineage cells were generated after neurospheres grafting, especially GDNF-T3/NSCs grafting, which greatly contributed to regenerating myelin, as indicated by a marked decrease in extent of demyelination after cells transplantation.

In addition, our results showed that the number of grafted cells in GDNF-T3/NSCs rats was higher than that in T3/NSCs and NSCs rats. Therefore, GDNF gene may increase survival of grafted NSCs in injury brain tissue, which is very crucial for improving therapeutic effect of NSCs for many CNS diseases.

In conclusion, we showed here that T3/NSCs, especially GDNF-T3/NSCs, more effectively improved functional outcome of EAE rats, alleviated the brain inflammatory infiltrations and demyelination, reduced astrogliosis, enhanced repopulation of neurons and oligodendrocytes, and increased expression of mRNA for oligodendrocyte lineage cells in brain compared to NSCs. These results suggest that T3/NSCs grafting, especially GDNF-T3/NSCs grafting, provides better neuroprotection for EAE than NSCs grafting alone.

## Figures and Tables

**Table 1:** The PCR sequences of the primers, sizes of the products, and the annealing temperatures.

Gene	Primer sequence (5′-3′)	Length (bp)	Annealing temperature (°C)
PDGF*α*R	F: CCAAATACTCCGACATCC	404	57
	R: CCAGAGCAGAACGCCATA		

GalC	F: CGGTGCCCTTGTTGTTGTG	252	59
	R: TGCCGTCTGTTGTTTGTCC		

MBP	F: TTCTTTAGCGGTGACAGGG	156	55
	R: GGAGCCGTAGTGGGTAGTT		

GAPDH	F: ACCACAGTCCATGCCATCAC	450	57
	R: TCCACCACCCTGTTGCTGTA		

**Table 2:** The reduction of infiltrates and improvement of myelin after neurospheres transplantation (mean ± SD, *n* = 5).

	Mean number of infiltrates	Mean number of cells/infiltrates	LFB density
Control	23.6 ± 4.3	57.4 ± 8.4	8.62 ± 0.56
NSCs	12.6 ± 3.1^*∗*^	42.0 ± 8.6^*∗*^	15.01 ± 1.45^*∗*^
T3/NSCs	9.2 ± 1.9^*∗*^	21.6 ± 5.9^*∗*#^	21.47 ± 2.03^*∗*#^
GDNF-T3/NSCs	5.80 ± 1.30^*∗*#^	12.2 ± 2.6^*∗*#*ψ*^	28.99 ± 1.39^*∗*#*ψ*^

^*∗*^Comparison with control group, *P* < 0.05; ^#^comparison with NSCs group, *P* < 0.05; ^*ψ*^comparison with T3/NSCs, *P* < 0.05.

**Table 3:** Differentiation of NSCs, T3/NSCs, and GDNF-T3/NSCs in vivo (mean ± SD, *n* = 5).

	BrdU^+^ (/mm^2^)	MAP2^+^/BrdU^+^ (%)	GalC^+^/BrdU^+^ (%)	GFAP^+^/BrdU^+^ (%)
NSCs	115.58 ± 19.34	16.35 ± 3.41	17.32 ± 5.98	49.01 ± 8.45
T3/NSCs	106.28 ± 12.5	25.03 ± 5.66^*∗*^	32.73 ± 4.65^*∗*^	26.58 ± 6.21^*∗*^
GDNF-T3/NSCs	135.60 ± 9.45^*∗*#^	43.67 ± 7.69^*∗*#^	36.93 ± 7.94^*∗*^	16.38 ± 4.14^*∗*#^

^*∗*^Comparison with NSCs group, *P* < 0.05; ^#^comparison with T3/NSCs, *P* < 0.05.

**Table 4:** The expression of endogenous astrocytes in lesion areas in each group (mean ± SD, *n* = 5).

	Normal	Control	NSCs	T3/NSCs	GDNF-T3/NSCs
Endogenous Astrocytes (/mm^2^)	22.40 ± 4.04	83.20 ± 7.46^*∗*^	53.40 ± 3.05^*∗*#^	43.20 ± 2.59^*∗*#*ψ*^	32.20 ± 3.11^*∗*#*ψ*Δ^

^*∗*^Comparison with normal group, *P* < 0.05; ^#^comparison with control group, *P* < 0.05; ^*ψ*^comparison with NSCs group, *P* < 0.05; ^Δ^comparison with T3/NSCs group, *P* < 0.05.

**Table 5:** Relative expression of PDGF*α*R, GalC, and MBP mRNA (PDGF*α*R/GAPDH, GalC/GAPDH, and MBP/GAPDH) (*n* = 5, mean ± SD).

	PDGF*α*R	GalC	MBP
Control	0.43 ± 0.07	0.40 ± 0.09	0.49 ± 0.07
NSCs	0.90 ± 0.11^*∗*^	0.74 ± 0.09^*∗*^	1.10 ± 0.14^*∗*^
T3/NSCs	1.42 ± 0.10^*∗*#^	1.50 ± 0.09^*∗*#^	1.43 ± 0.10^*∗*#^
GDNF-T3/NSCs	1.52 ± 0.09^*∗*#^	1.69 ± 0.08^*∗*#*ψ*^	1.62 ± 0.10^*∗*#*ψ*^

^*∗*^Comparison with control group, *P* < 0.05; ^#^comparison with NSCs group, *P* < 0.05; ^*ψ*^comparison with T3/NSCs, *P* < 0.05.
